# A unique DNA methylation signature defines a population of IFN-γ/IL-4 double-positive T cells during helminth infection

**DOI:** 10.1002/eji.201344098

**Published:** 2014-03-25

**Authors:** Aimée M Deaton, Peter C Cook, Dina De Sousa, Alexander T Phythian-Adams, Adrian Bird, Andrew S MacDonald

**Affiliations:** 1 Wellcome Trust Centre for Cell Biology, University of EdinburghEdinburgh, UK; 2 Institute of Immunology and Infection Research, Centre for Immunity, Infection and Evolution, University of EdinburghEdinburgh, UK

**Keywords:** DNA methylation, Gata3, Helminth, Th1/Th2

## Abstract

Th1 and Th2 cell fates are traditionally viewed as mutually exclusive, but recent work suggests that these lineages may be more plastic than previously thought. When isolating splenic CD4^+^ T cells from mice infected with the parasitic helminth *Schistosoma mansoni*, we observed a defined population of IFN-γ/IL-4 double-positive cells. These IFN-γ^+^IL-4^+^ cells showed differences in DNA methylation at the *Ifng* and *Il4* loci when compared with IFN-γ^+^IL-4^−^ (Th1) and IFN-γ^−^IL-4^+^ (Th2) cells, demonstrating that they represent a distinct effector cell population. IFN-γ^+^IL-4^+^ cells also displayed a discrete DNA methylation pattern at a CpG island within the body of the *Gata3* gene, which encodes the master regulator of Th2 identity. DNA methylation at this region correlated with decreased Gata3 levels, suggesting a possible role in controlling Gata3 expression. These data provide important insight into the molecular mechanisms behind the co-existence of Th1 and Th2 characteristics.

## Introduction

Th1 and Th2 cells are specialized subsets of CD4^+^ T helper cells that respond to different modes of infection and which express the signature cytokines IFN-γ and IL-4, respectively. Transcription factors are involved in specifying Th1 and Th2 cell fate: T-bet is responsible for Th1 lineage determination while Gata3 is the key factor involved in Th2 development 1,2. Although Th1 and Th2 are generally thought of as mutually exclusive cell fates, since factors made by one cell type antagonize the generation of the other 1,3, several studies suggest some flexibility in Th1 and Th2 cell identity. Th1 and Th2 cytokines can be co-expressed by T-cell clones in vitro 4,5, and in vitro generated Th2 cells can be reprogrammed by Th1-promoting lymphocytic choriomeningitis virus to adopt a Gata3^+^T-bet^+^ phenotype 6. Similarly, in vitro derived Th1 cells can be converted to IL-4-producing Th2 cells 7, and T cells with both Th1 and Th2 characteristics can be found during helminth infection 8,9. Importantly, the molecular details of how CD4^+^ T cells can stably maintain both Th1 and Th2 characteristics remain unclear.

Epigenetic mechanisms, including DNA methylation occurring at the dinucleotide sequence CpG, can be involved in maintaining Th1 and Th2 identity 10,11. When DNA methylation occurs at gene promoters, it is associated with transcriptional silencing 12,13. In general, the *Ifng* locus shows reduced DNA methylation upon Th1 differentiation, while the *Il4/Il5/Il13* locus loses DNA methylation upon Th2 differentiation. This is consistent with upregulation of *Ifng* expression during Th1 differentiation and induction of *Il4* and other Th2 cytokines during Th2 differentiation 10,11.

In vertebrates, the genome is punctuated by CpG islands (CGIs), which have an increased density of CpG dinucleotides compared to the rest of the genome and an elevated G+C base composition 14. Although CGIs are usually unmethylated, DNA methylation can occur during normal development 13. CGIs frequently associate with gene promoters, although they also occur within and between annotated genes 15. We recently carried out a genome-wide survey of DNA methylation at CGIs in immune cells and identified just one CGI methylation difference between Th1 and Th2 cells differentiated in vitro. This occurred at a CGI within the body of the gene encoding Gata3, the master regulator of Th2 cell identity 16.

We wanted to investigate DNA methylation of *Ifng* and *Il4* in a physiologically relevant infection setting. As Gata3 regulates Th2 differentiation, we isolated CD4^+^ T cells from mice infected with the Th2-inducing parasitic helminth *Schistosoma mansoni* 17. We observed splenocytes positive for both IFN-γ and IL-4 in infected mice, in addition to conventional Th1 and Th2 cells producing only IFN-γ or IL-4. The IFN-γ^+^IL-4^+^ cells displayed a distinct DNA methylation signature at key cytokine genes and at *Gata3*, suggesting that methylation patterns may be important for allowing the stable co-existence of Th1 and Th2 phenotypes. Our data further challenge the paradigm that Th1 and Th2 cell fates are mutually exclusive by demonstrating that IFN-γ^+^IL-4^+^ cells are a discrete cell population, different from Th1 and Th2 cells with respect to DNA methylation as well as cytokine production. Our data also suggest a potential role for the *Gata3* CGI in regulating Gata3 expression and highlight possible regulatory significance for intragenic CGI methylation more generally.

## Results and discussion

### IFN-γ^+^IL-4^+^ cells are generated during *S. mansoni* infection

**Figure 1:**
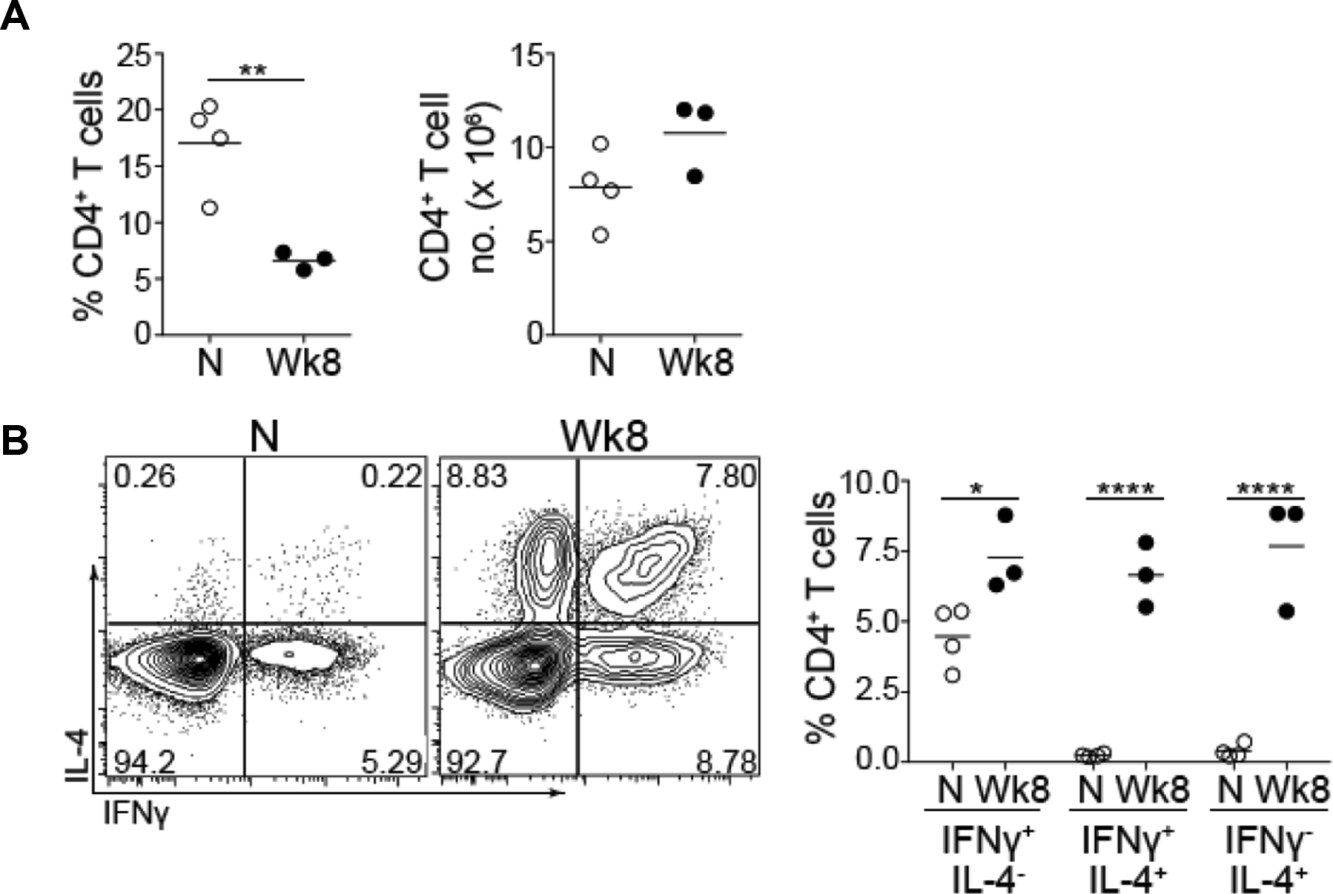
IFN-γ^+^IL-4^+^ cells are generated during *Schistosoma mansoni* infection. Splenocytes were isolated from infected mice taken from the same experiment (Wk 8) or uninfected age-matched controls (N). (A) The proportion and number of splenic CD4^+^ T cells was assessed by flow cytometry. Each symbol represents an individual animal and the mean of each sample group is shown as a horizontal line. Statistical significance was assessed using a Student's *t*-test and data are representative of two independent experiments. (B) Purified splenic CD4^+^ T cells were stimulated with PMA, ionomycin, and GolgiStop. FACS plots show intracellular staining of IFN-γ and IL-4. Dead cells, doublets and TCR-β^−^CD4^−^ cells were excluded prior to assessing cytokine expression (Supporting Information Fig. 1). Plots are representative of five independent infections where either pools of spleens were analyzed or animals were analyzed separately. Results were similar in both cases. The percentage of splenic CD4^+^ cells expressing IFN-γ alone, both IFN-γ and IL-4, or IL-4 alone for individual mice taken from the same experiment are plotted on the right hand side of the figure, significance was assessed using two-way ANOVA. *p < 0.05, **p < 0.01 and ****p < 0.0001.

In order to examine DNA methylation in an in vivo infection setting we isolated splenic CD4^+^ T cells from mice that had been infected with *S. mansoni* for 8 weeks and from age-matched uninfected controls (Fig.1A). A marked proportion of CD4^+^ T cells displayed properties of both Th1 and Th2 cells in that they simultaneously made both IFN-γ and IL-4 8 (Fig.1B and Supporting Information Fig. 1). Conventional IFN-γ^+^IL-4^−^ Th1 cells and IFN-γ^−^IL-4^+^ Th2 cells were also present, consistent with previous reports 18,19 and CD4^+^ T cells from uninfected mice showed significantly less expression of IFN-γ or IL-4 (Fig.1B). IFN-γ^+^IL-4^+^ cells were observed in five separate *S. mansoni* infections with the proportion varying from approximately 2–9% of CD4^+^ T cells (data not shown), demonstrating that IFN-γ^+^IL-4^+^ cells can be found in the spleen in a Th2-dominated infection setting.

A balance between Th1 and Th2 responses is critical for host survival in *S. mansoni* infection 17. The Th2 response is crucial for limiting disease in the early stages of the infection 20, while prolonged or excessive Th2 responses lead to liver fibrosis and decreased survival, mediated predominantly by IL-13 21. IFN-γ may help to counter-regulate such Th2-mediated fibrotic disease during infection 22–24. Thus, IFN-γ^+^IL-4^+^ double positive cells may help maintain a balance between extreme Th1 and Th2 polarization during *S. mansoni* infection.

### IFN-γ^+^IL-4^+^ cells show a distinct DNA methylation pattern at cytokine gene loci and *Gata3*

**Figure 2:**
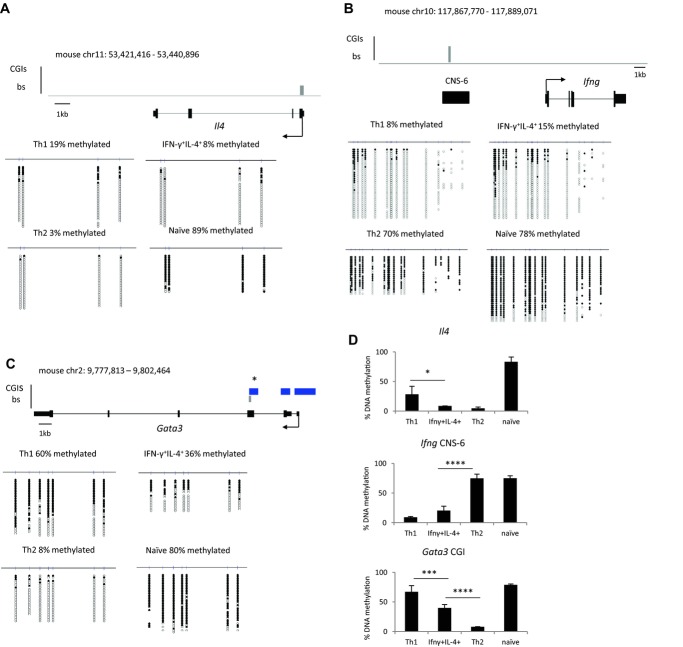
IFN-γ^+^IL-4^+^ cells show a distinct DNA methylation signature at *Il4, Ifng* and *Gata3*. (A) *Il4* promoter, (B) *Ifng* CNS-6, (C) *Gata3* gene body CGI (*) DNA methylation was determined using bisulfite sequencing. The relevant gene and the region analyzed by bisulfite (grey bar) are shown (upper panel). The arrow indicates the origin and direction of transcription and, where present, CGIs are represented by blue bars. Bisulfite sequencing data for Th1, Th2, IFN-γ^+^IL-4^+^ CD4^+^ T cells isolated from infected mice and naïve CD4^+^ T cells isolated from uninfected controls are also shown (lower panel). Filled circles represent methylated CpG residues, empty circles represent unmethylated CpGs and each row corresponds to an individual sequenced clone. (A–C) Data shown are from single experiments representative of two independent experiments performed. (D) The percentage DNA methylation is shown as mean + SD of two independent experiments. Statistical significance of methylation differences between Th1, Th2, and IFN-γ^+^IL-4^+^ cells was assessed using QUMA software and Mann–Whitney *U* test, * *p* < 0.05, *** *p* < 0.001 and **** *p* < 0.0001.

To examine whether IFN-γ^+^IL-4^+^ cells are distinct on a molecular level from Th1 and Th2 cells, we examined DNA methylation of key immune system genes. IFN-γ^+^IL-4^−^ (Th1), IFN-γ^−^IL-4^+^ (Th2), and IFN-γ^+^IL-4^+^ cell populations were FACS sorted, with purities of 96.8–98% (Supporting Information Fig. 2), prior to methylation analysis by bisulfite genomic sequencing. DNA from the isolated cells was treated with sodium bisulfite, which converts unmethylated cytosines to uracil but leaves methylated cytosines unconverted, then PCR was performed to amplify genomic regions of interest, which were subsequently sequenced 25. First we examined DNA methylation at the key cytokine gene loci, *Ifng* and *Il4*, which have been reported to differ in methylation status between in vitro generated Th1 and Th2 cells 10. This revealed a distinct DNA methylation signature for IFN-γ^+^IL-4^+^ cells compared with conventional Th1 and Th2 cells. In double positive cells, both the *Il4* promoter and the *Ifng* CNS-6 regulatory region showed significant demethylation (Fig.2A and B). Conventional Th1 and Th2 cells lacked methylation at the locus for their signature cytokine while the locus for the opposing cytokine was more extensively methylated. In CD4^+^ cells isolated from uninfected mice both *Il4* and *Ifng* were completely methylated (Fig.2A and B). DNA methylation is frequently associated with gene repression and these results are broadly consistent with the fact that Th1 cells do not express *Il4*, Th2 cells do not express *Ifng*, while IFN-γ^+^IL-4^+^ cells, which have low levels of DNA methylation at *Il4* and *Ifng*, express both genes. However, it is worth noting that in Th1 cells the *Il4* promoter showed a dramatic decrease in DNA methylation compared with naïve controls (Fig.2A). This could suggest that demethylation of the *Il4* locus is a general feature of CD4^+^ T cells in Th2 environments. Nevertheless, our data demonstrate that ex vivo IFN-γ^+^IL-4^+^, Th1, and Th2 cells are distinct from each other with respect to DNA methylation as well as cytokine production. During *S. mansoni* infection, the spleen is an accepted site for assessing responding lymphocytes, which include circulating effector and effector/memory CD4^+^ T cells 26. An important next step in our studies will be to assess the methylation signature of IFN-γ^+^IL-4^+^ T cells isolated from effector sites such as the liver.

We have previously shown that the only CGI methylation difference between in vitro differentiated Th1 and Th2 cells occurs at a CGI in the body of the *Gata3* gene, overlapping its third exon (Fig.2C) 16. We assessed DNA methylation of this region in the different CD4^+^ T-cell populations isolated ex vivo from *S. mansoni* infection. IL-4-producing Th2 cells showed almost complete demethylation of the *Gata3* CGI (8% methylation). In contrast, Th1 cells were heavily methylated at this CGI (60% methylation). IFN-γ^+^IL-4^+^ cells had *Gata3* DNA methylation levels intermediate between those of Th1 and Th2 cells (36% methylation, Fig.2C). Examination of a different bisulfite PCR amplicon within the *Gata3* gene body CGI yielded similar results (Supporting Information Fig. 3A and B). For all regions interrogated, two independent experiments gave comparable DNA methylation levels in each of the cell populations (Fig.2D).

The distinct DNA methylation patterns observed at key immune gene loci in IFN-γ^+^IL-4^+^ cells demonstrate that these cells are different on a molecular level from Th1 and Th2 cells and therefore represent a distinct CD4^+^ T cell population generated during *S. mansoni* infection. Furthermore, this unique DNA methylation signature may represent an epigenetic state that allows for inherent flexibility in the identity of these double positive cells.

### Methylation of the Gata3 gene body CGI negatively correlates with gene expression

**Figure 3:**
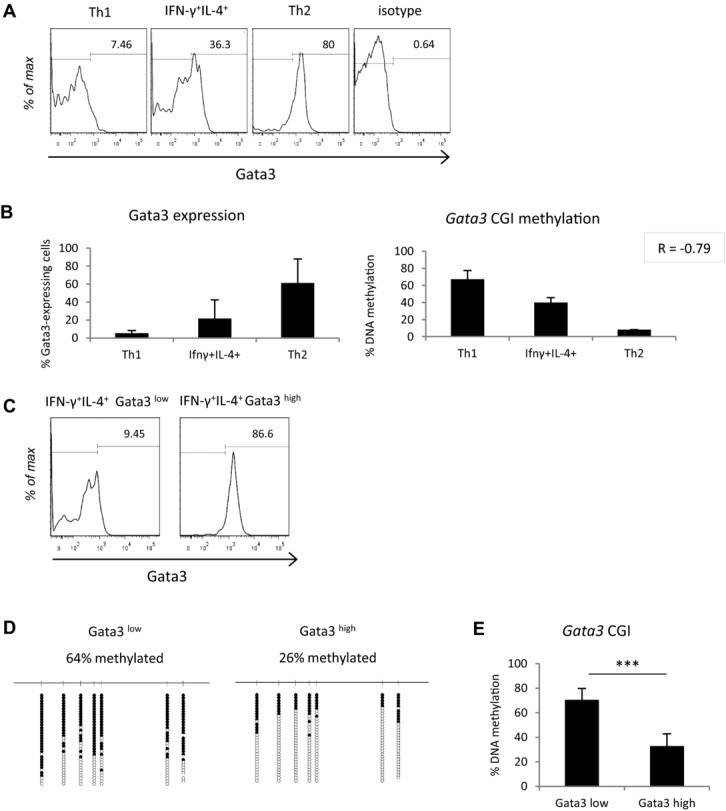
*Gata3* gene body CGI methylation negatively correlates with gene expression. (A) Gata3 expression as assessed by flow cytometry in Th1, Th2, and IFN-γ^+^IL-4^+^ cells. (B) The percentage of Gata3-expressing cells in spleens isolated from *S. mansoni* infection is plotted alongside the overall percentage DNA methylation, as assessed by bisulfite sequencing, at the *Gata3* CGI for each cell population. Pearson's correlation coefficient (R) for the two datasets is shown in top right hand corner. (C) Sorting of IFN-γ^+^IL-4^+^ cells into Gata3^low^ and Gata3^high^ cells by FACS. (D) *Gata3* methylation of Gata3^low^ and Gata3^high^ cells as quantified by bisulfite sequencing, labelling as in Fig.2. (E) Mean *Gata3* gene body CGI methylation levels in Gata3^low^ and Gata3^high^ cells. Statistical significance was assessed using QUMA software and Mann–Whitney *U* test, ***p < 0.001. (A, C, D) Data shown are from a single experiment, representative of two independent experiments performed. (B, E) Data are shown as mean + SD of two independent experiments. FACS experiments are gated on FSC-A and SSC-A, FSC-A and FSC-W, CD4 expression, and lastly on IFN-γ and IL-4 production.

It is well established that DNA methylation of a promoter CGI correlates with silencing of the associated gene 12,13, but the effect of gene body CGI methylation on transcription is less clear. We assessed Gata3 expression using intracellular staining and FACS in Th1, Th2, and IFN-γ^+^IL-4^+^ cells and compared Gata3 levels to DNA methylation levels within each population (Fig.3A and B). Methylation of the *Gata3* intragenic CGI correlated with decreased expression of Gata3, despite the fact that this CGI is not located at the gene's promoter (Fig.3B). For example, in Th1 cells where this CGI is heavily methylated, only 7% of cells stained positive for Gata3. In contrast, in Th2 cells where *Gata3* is demethylated, 80% of cells expressed Gata3. IFN-γ^+^IL-4^+^ cells showed Gata3 expression intermediate between Th1 and Th2 cells, consistent with their intermediate DNA methylation levels (Fig.3A and B). Moderate Gata3 expression and gene body methylation levels in IFN-γ^+^IL-4^+^ cells may be important for allowing them to possess both Th1 and Th2 characteristics.

We then examined the relationship between *Gata3* methylation and gene expression more closely within the IFN-γ^+^IL-4^+^ population. IFN-γ^+^IL-4^+^ cells were FACS sorted into two additional populations: those that expressed high levels of Gata3 and those that expressed low levels (Fig.3C and Supporting Information Fig. 4A). DNA methylation at the *Gata3* intragenic CGI was then assessed by bisulfite sequencing. Gata3^low^ cells displayed a significantly higher overall level of DNA methylation than Gata3^high^ cells (64% compared with 26%, *p* ≤ 0.0005) with approximately half of the DNA molecules in the Gata3^low^ cells methylated at all sites tested (Fig.3D and E). This is consistent with an association between gene body CGI methylation and transcriptional repression in an infection setting. Further, as expected, Gata3^high^ cells showed moderately higher levels of IL-4 expression, and lower levels of IFN-γ expression, when compared with Gata3^low^ cells (Supporting Information Fig. 4B).

*Gata3* possesses a promoter CGI in addition to its gene body CGI (Fig.2C) 16. Notably, DNA methylation analysis of the promoter CGI showed that it remained unmethylated in all cell populations despite differences in *Gata3* expression (Supporting Information Fig. 3C). This finding highlights a potential role for the *Gata3* gene body CGI rather than the promoter CGI in regulating *Gata3* gene expression via DNA methylation. It is possible that the gene body CGI is an alternative promoter for *Gata3*, or acts as another kind of regulatory element, such as an enhancer, that functions only when it is demethylated. Another explanation could be that gene body CGI methylation inhibits transcriptional elongation and therefore its removal facilitates *Gata3* expression 27.

*Tbx21*, the locus from which T-bet is expressed, has a promoter CGI but no gene body CGI 15. Analysis of existing DNA methylation data for naïve CD4^+^ cells and in vitro differentiated Th1 and Th2 cells showed that the *Tbx21* promoter CGI is completely unmethylated in all of these cell types (16, and data not shown). Based on this, as well as the lack of methylation at the *Gata3* promoter (Supporting Information Fig. 3C), we would suggest that it is unlikely that the DNA methylation status of *Tbx21* changes in CD4^+^ T cells from *S. mansoni* infection. However, it is possible that expression of T-bet along with expression of Gata3 contributes to the dual identity of IFN-γ^+^IL-4^+^ cells, even if it is not associated with differences in DNA methylation.

The nature of the regulatory role played by the *Gata3* gene body CGI and whether DNA demethylation of this CGI is a cause or consequence of *Gata3* expression warrants further investigation. Irrespective, our results indicate that intragenic CGI methylation might be more reflective of transcriptional activity generally than DNA methylation at promoter CGIs, as these are almost always unmethylated 15,28.

### Concluding remarks

In summary, we have identified a population of splenic CD4^+^ T cells generated during *S. mansoni* infection, which expresses both IFN-γ and IL-4 simultaneously. These IFN-γ^+^IL-4^+^cells are molecularly distinct from Th1 and Th2 cells as they show a unique DNA methylation signature at key immune genes, suggesting that DNA methylation is important for allowing co-existence of Th1 and Th2 characteristics. We have also uncovered a relationship between methylation of a CGI in the body of *Gata3* and repression of Gata3 expression. This raises the interesting possibility that intragenic CGI methylation may represent a novel general mechanism of gene regulation in immune cells.

## Materials and methods

### Animals and S. mansoni infection

Experiments were performed using female C57BL/6 mice, which were maintained under specific pathogen-free conditions and used at 8–12 weeks of age. Experiments were conducted under a Project License granted by the Home Office (United Kingdom) in accordance with local guidelines. Mice were infected percutaneously with 40–80 *S. mansoni* cercariae, as previously described 29. Infections were allowed to proceed for 8 weeks prior to culling and spleen cell isolation.

### T-cell isolation, culture, and FACS

CD4^+^ T cells were positively or negatively selected from naïve and infected spleens (Miltenyi Biotec or Life Technologies) and cultured in X-Vivo 15 medium (Lonza, BioWhittaker) supplemented with l-glutamine and 2-mercaptoethanol, and then stimulated with PMA (10 ng/mL), ionomycin (1 μg/mL), and Golgistop (BD Biosciences; 1:1000) for 4–5 hours at 37°C, 5% CO_2_. Cells were fixed, permeabilized and stained with antibodies against IFN-γ (Biolegend), IL-4 (BD Biosciences), and Gata-3 (BD Biosciences). For DNA methylation experiments, 6–10 spleens were pooled in order to obtain adequate numbers of cells for downstream analysis. Sorting was carried out using a BD FACS Aria. For statistical analysis, GraphPad Prism software was used to perform Student's *t*-test or ANOVA where appropriate.

### Bisulfite genomic sequencing

DNA was isolated from sorted cells according to standard protocols. Bisulfite treatment and sequencing was performed as previously described 30. Statistical analysis was carried out using the QUMA package 31 and statistical significance was assessed by Mann–Whitney *U* test. Sequences of primers used for PCR amplification of bisulfite treated DNA are available upon request.

## Abbreviations

CGI: CpG island
