# Supplementary material for: A unique DNA methylation signature defines a population of IFN-γ/IL-4 double-positive T cells during helminth infection
Source: Eur J Immunol. 2014 Mar 25;44(6):1835–41. doi: 10.1002/eji.201344098 (PMC4231227; doi:10.1002/eji.201344098)
Supplement: Supplementary file 2 [file eji0044-1835-sd2.pdf]

# European Journal of Immunology

## Supporting Information

## for

## DOI 10.1002/eji.201344098


Aimée M. Deaton, Peter C. Cook, Dina De Sousa, Alexander T. Phythian-Adams, Adrian Bird and Andrew S. MacDonald

**A unique DNA methylation signature defines a population of IFN-$\gamma$/IL-4 double-positive T cells during helminth infection**

© 2014 WILEY-VCH Verlag GmbH & Co. KGaA, Weinheim

Supporting Information

Figure 1

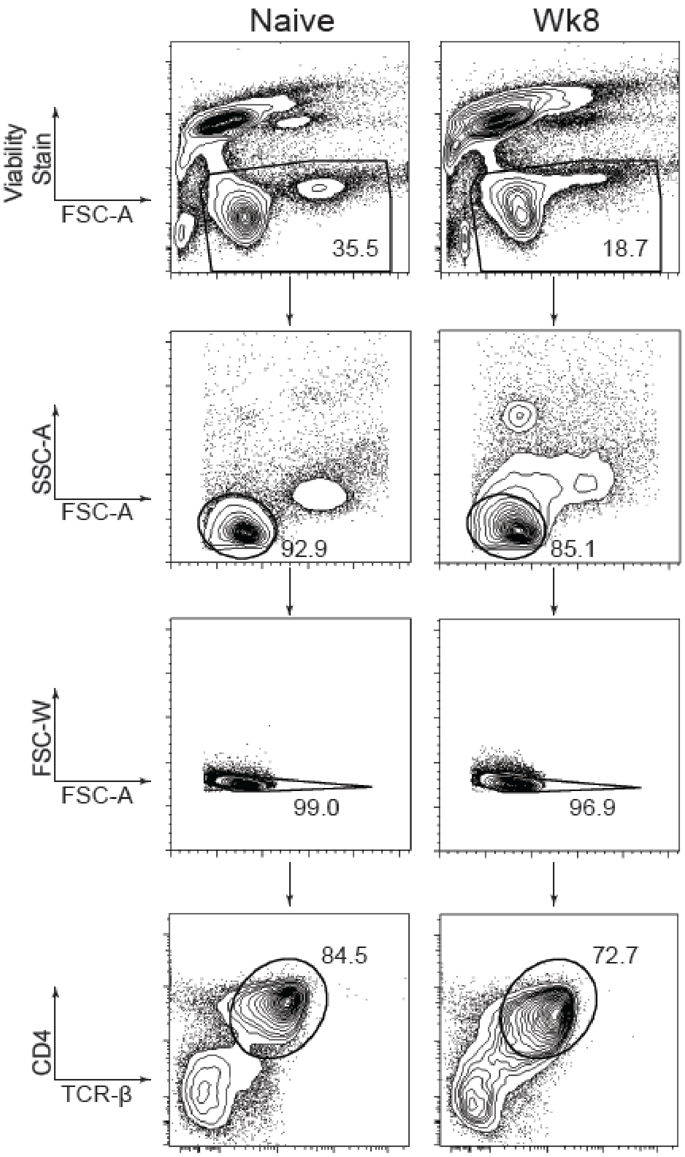

Supporting Information

Figure 2

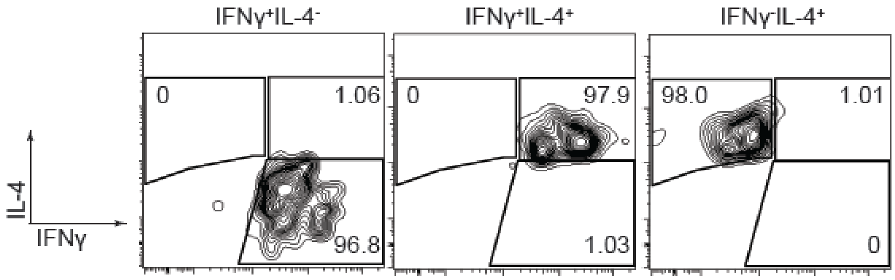

# Supporting Information

# Figure 3

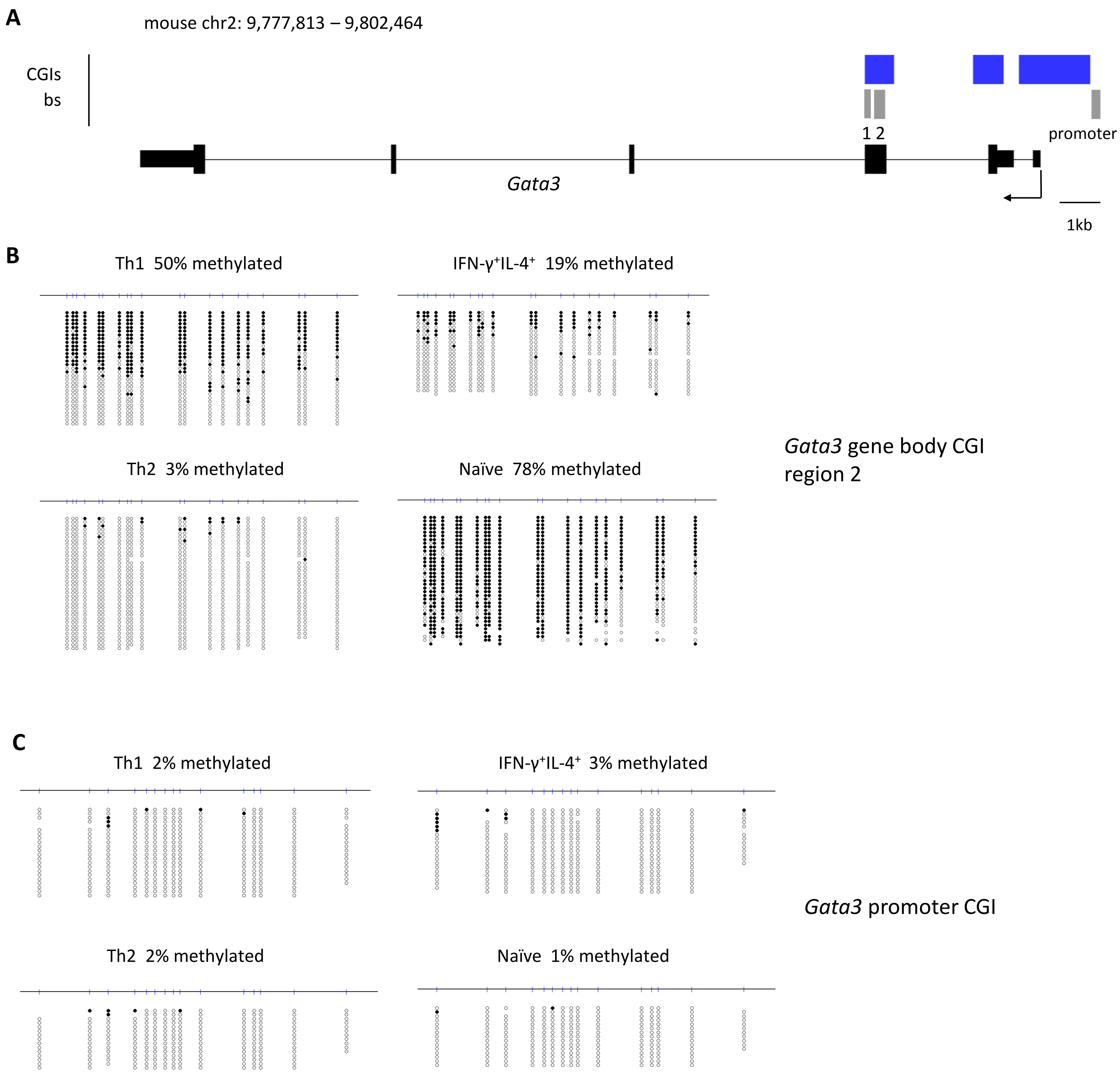

Supporting Information

Figure 4

**A**

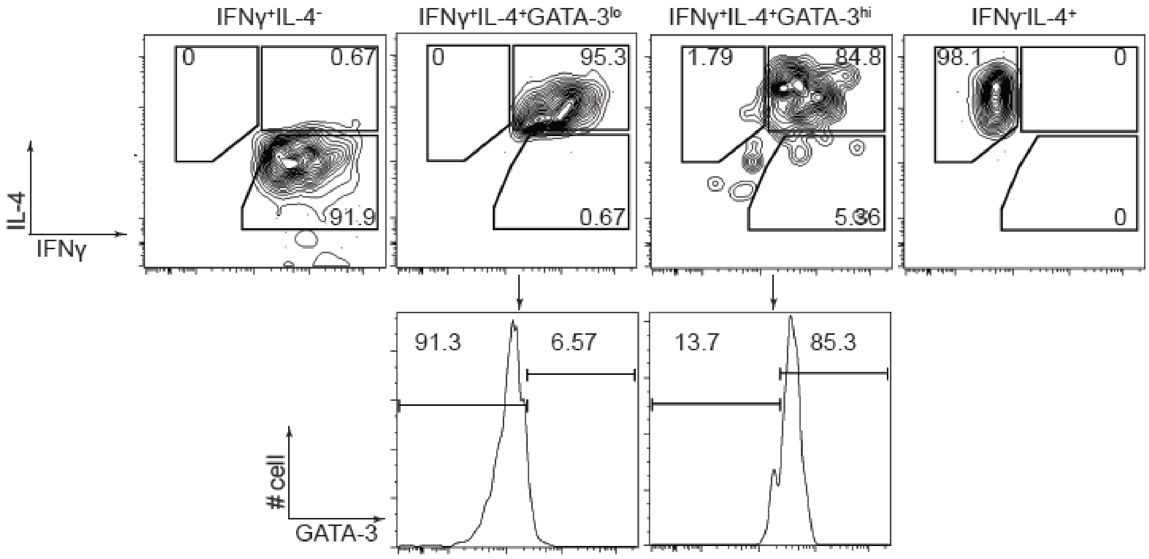


**B**

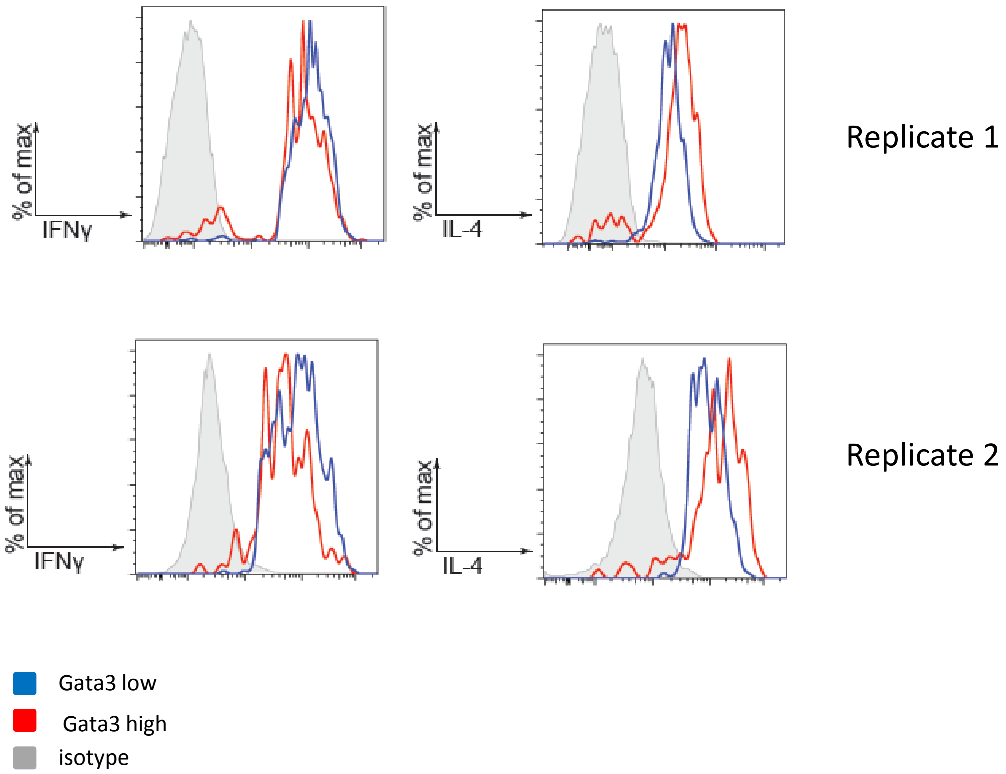

Supporting Information - Figure Legends

Figure 1: Gating strategy for identification of IFN-$\gamma^+$IL-$4^+$ cells used in Figure 1B.

C57BL/6 mice were infected with 40-80 *S. mansoni* cercariae percutaneously and spleens harvested 8 weeks later. Splenocytes were enriched for CD4+ cells and stimulated with PMA, Ionomycin and GolgiStop. Representative FACS panels show the gating strategy for generating plots in Figure 1B. Dead cells, doublets and TCR-$\beta^-$ CD$4^-$ cells were excluded prior to assessing IFN-$\gamma$ and IL-4 expression.

Figure 2: Purities of FACS sorted cells used for methylation analysis.

Representative sorts of Th1 (IFN-$\gamma^+$), Th2 (IL-$4^+$) and IFN-$\gamma^+$IL-$4^+$ CD$4^+$ T cells used for methylation analysis in Figure 2. Gating was carried out as in Supporting Information Figure 1.

Figure 3: DNA methylation analysis of *Gata3* gene body and promoter CGIs

(A) Diagram of *Gata3* showing the position of CGIs (blue bars) and regions analyzed by bisulfite (grey bars). The gene body CGI regions are labelled “1” and “2” and the promoter CGI region analyzed is labelled “promoter”. Bisulfite sequencing results are shown for; (B) *Gata3* gene body CGI region 2 and (C) *Gata3* promoter in Th1, Th2 and IFN-$\gamma^+$IL-$4^+$ CD$4^+$ T cells isolated from infected mice and naïve CD$4^+$ T cells isolated from uninfected controls. Filled circles represent methylated CpG residues, empty circles represent unmethylated CpGs and each row corresponds to an individual clone. Data are representative of two independent experiments.

Figure 4: Gata3$^{high}$ cells display higher IL-4 expression and lower IFN-γ expression compared to Gata3$^{low}$ cells.

(A) Representative sorts of IFN-γ$^{+}$IL-4$^{+}$ Gata3$^{low}$ and Gata3$^{high}$ cells used for methylation analysis. Gating was carried out as in Supporting Information Figure 1. (B) Analysis of IFN-γ and IL-4 expression in sorted Gata3$^{low}$ and Gata3$^{high}$ cell populations. Gata3$^{low}$ cells are shown in blue, Gata3$^{high}$ cells in red, and isotype controls in grey. Two replicate experiments are shown.
