# Supplementary material for: A unique DNA methylation signature defines a population of IFN-γ/IL-4 double-positive T cells during helminth infection
Source: Eur J Immunol. 2014 Mar 25;44(6):1835–41. doi: 10.1002/eji.201344098 (PMC4231227; doi:10.1002/eji.201344098)
Supplement: Supplementary file 1 [file eji0044-1835-sd1.pdf]

European Journal of Immunology

Peer review correspondence

A unique DNA methylation signature defines a population of IFN-γ/IL-4 double-positive T cells during helminth infection

Aimée M. Deaton, Peter C. Cook, Dina De Sousa, Alexander T. Phythian-Adams, Adrian Bird and Andrew S. MacDonald.

Corresponding author: Andrew S. MacDonald., Manchester Collaborative Centre for Inflammation Research

---

Review Timeline:	Submission date:	19 September 2013
	First editorial decision:	21 October 2013
	First revision received:	16 January 2014
	Second editorial decision:	10 February 2014
	Second revision received:	14 February 2014
	Accepted:	20 February 2014

Handling Executive Committee member: Prof. Shimon Sakaguchi

Please note that the correspondence below does not include the standard editorial instructions regarding preparation and submission of revised manuscripts, only the scientific revisions requested and addressed.

First Editorial Decision – 21 October 2013

Dear Dr. MacDonald,

Manuscript ID eji.201344098 entitled "A unique DNA methylation signature defines a population of IFNγ/IL-4 double positive T cells during helminth infection." which you submitted to the European Journal of Immunology has been reviewed. The comments of the referees are included at the bottom of this letter.

A revised version of your manuscript that takes into account the comments of the referees will be reconsidered for publication.

You should also pay close attention to the editorial comments included below. *In particular, please edit your figure legends to follow Journal standards as outlined in the editorial comments. Failure to do this will result in delays in the re-review process.*

European Journal of Immunology

Please note that submitting a revision of your manuscript does not guarantee eventual acceptance, and that your revision will be re-reviewed by the referees before a decision is rendered.

If the revision of the paper is expected to take more than three months, please inform the editorial office. Revisions taking longer than six months may be assessed by new referees to ensure the relevance and timeliness of the data.

Once again, thank you for submitting your manuscript to European Journal of Immunology and we look forward to receiving your revision.

Yours sincerely,
Karen Chu

On behalf of Prof. Shimon Sakaguchi

Dr. Karen Chu
Editorial Office
European Journal of Immunology
e-mail: ejied@wiley.com

****

Reviewer: 1
Comments to the Author
This paper by Deaton et al. reported that IFNγ/IL-4 double-producing CD4 T cells are generated after S. mansoni infection, the DNA methylation signature at the ifng and il4 gene loci of the IFNγ+IL-4+ CD4 T cells is distinct from that of IFNγ or IL-4 single-producing cells, the level of Gata3 expression in IFNγ+IL-4+ cells is intermediate compared to that of Th2 and Th1 cells and that the level of Gata3 expression negatively correlates with the DNA methylation at the CpG island (CGI) on the gata3 gene locus in IFNγ+IL-4+ CD4 T cells. The authors suggest that the modification of intragenic CGI methylation is a new mechanism of cytokine gene regulation in effector CD4 T cells. Although their findings are interesting, several major points need to be addressed:

(1) In Figure 1, the authors should carefully analyze the population of IFNγ+IL-4+ CD4 T cells after S. mansoni infection. There is a possibility that the IFNγ+IL-4+ CD4 T cells might be doublet cells or dead cells, because these cells are on the diagonal line in Figure 1B. The authors should test the population of IFNγ+IL-4+ CD4 T cells after removing doublet cells and dead cells by using the combination of FSC-W and SSC-W, and dead cell staining reagents.

European Journal of Immunology

(2) In Figures 2 and S1, the authors showed that the methylation of the il4, ifng and gata3 gene loci negatively correlated with the gene expression levels. Naïve CD4 T cells cannot produce more than the amount of IFNγ and IL-4 from Th1 and Th2 cells, respectively, so it is reasonable for naïve CD4 T cells to maintain the promoters of the il4 gene and CNS-6 of the ifng gene methylated. However, although naïve CD4 T cells are known to express Gata3, the gene body of Gata3 is methylated in naïve CD4 T cells, as well as in Th1 cells. This result does not fit their conclusions. The authors should discuss this discrepancy. It might be helpful to investigate the chromatin modifications, H3K4 methylation, H3K9 methylation and H3K14 acetylation at the target regions.

(3) In Figure 3C, the authors separated IFNγ+IL-4+ CD4 T cells into two types of cells based on the Gata3 expression level; high and low. In Figure 3D, they showed that the methylation signature at the CGI of the gata3 gene locus in Gata3-high cells was lower than that in Gata3-low cells. The authors should reanalyze the Gata3 expression and IL-4/IFNγ production, and show whether the level of Gata3 expression correlates positively with IL-4 and negatively with IFNγ.

Reviewer: 2

Comments to the Author

Deaton et al. describe IFN-g/IL-4 co-producing CD4 T cells in spleens of S. mansoni-infected mice. These cells exhibit distinct DNA methylation levels at the Ifng, Il4 and Gata3 loci in between those of IFN-g+IL-4- and IFN-g-IL-4+ cells from the same mice. The IFN-g+IL-4+ population contains GATA-3 low and high cells. GATA-3 low cells display stronger DNA methylation than GATA-3 high cells at intragenic but not promoter sites of the Gata3 gene, suggesting that intragenic DNA methylation may be indicative of transcription levels.

The intermediate DNA methylation levels at key Th1 and Th2 gene loci in IFN-g+IL-4+ cells are an interesting correlative observation. It may reflect one mechanism that underlies the stable phenotype of this IFN-g+IL-4+ T cell population. However, the current manuscript has several aspects that require major revisions:

1.) Why do the authors claim that their finding of IFN-g+IL-4+ T cells upon infection with S. mansoni is “surprising” (p. 5, l. 15)? It should be mentioned that this phenotype has already been described [1-3], also in response to S. mansoni [4] and other helminth infections [5].

2.) In Fig. 1B, a control showing IFN-g vs. IL-4 production of PMA/ionomycin-restimulated CD4 cells from uninfected mice is required. These uninfected mice should be of a similar age as the infected animals to get an idea of the CD4 memory component independent of the infection with S. mansoni (cf. next point). The control without restimulation is not sufficient.

European Journal of Immunology

3.) The cells were neither restimulated in an antigen-specific manner nor taken from an effector organ such as the liver. Thus, the analyzed populations were rather undefined, most likely being a mixture of S. mansoni-specific effectors and other effector/memory cells with unknown differentiation history. This limitation should at least be critically discussed.

4.) A purity of 75%-96% is rather low for a FACS sort. May this explain why in Fig. 2A the Th1 cells showed strong demethylation at the Il4 gene compared with the naive control or may this result from their undefined differentiation history as discussed above? The authors should comment on this finding instead of just claim that "in conventional Th1 and Th2 cells only the locus for the signature cytokine lacked methylation" (p. 6, l. 23). The sort pictures should be included as a supplementary figure including the population that was only 75% pure to better define the contaminating cell fraction.

5.) IFN-g+IL-4+ cells exhibit long-term co-expression of the Th1 and Th2 lineage-specifying transcription factors T-bet and GATA-3, respectively. Why do the authors only analyze DNA methylation of the Gata3 locus? Data on the Tbx21 locus should be included as well.

6.) The selection of only one region at each gene for the DNA methylation analysis seems a bit arbitrary and requires explanation. The authors should at least show data for the promoter and one key regulatory element of each gene and maybe comment on further ones.

7.) For Fig. 2 and Fig. 3, the results of the second experiment should be shown, perhaps as bar diagrams with error bars, normalized to the naive controls.

8.) How do the authors interpret that the IFN-g+IL-4+ population contained a GATA-3 low compartment that shows GATA-3 expression levels and DNA methylation like Th1 cells? Does this contradict their claim of a distinct population?

9.) The site of the GATA-3 gene body CGI should be specified. Is it located in an exon?

10.) How is the differential DNA methylation at the GATA-3 gene body CGI related to the IFN-g/IL-4 co-expressing T cells? The authors should better connect these two parts.

1. Kelso A, Gough NM (1988) Coexpression of granulocyte-macrophage colony-stimulating factor, gamma interferon, and interleukins 3 and 4 is random in murine alloreactive T-lymphocyte clones. Proc Natl Acad Sci U S A 85: 9189-9193.

2. Paliard X, de Waal Malefijt R, Yssel H, Blanchard D, Chretien I, et al. (1988) Simultaneous production of IL-2, IL-4, and IFN-gamma by activated human CD4+ and CD8+ T cell clones. J Immunol 141: 849-855.

3. Firestein GS, Roeder WD, Laxer JA, Townsend KS, Weaver CT, et al. (1989) A new murine CD4+ T cell subset with an unrestricted cytokine profile. J Immunol 143: 518-525.

4. Lohning M, Grogan JL, Coyle AJ, Yazdanbakhsh M, Meisel C, et al. (1999) T1/ST2 expression is enhanced on CD4+ T cells from schistosome egg-induced granulomas: analysis of Th cell cytokine coexpression ex vivo. J Immunol 162: 3882-3889.

5. Peine M, Rausch S, Helmstetter C, Frohlich A, Hegazy AN, et al. (2013) Stable T-bet(+)GATA-3(+) Th1/Th2 Hybrid Cells Arise In Vivo, Can Develop Directly from Naive Precursors, and Limit Immunopathologic Inflammation. PLoS Biol 11: e1001633.

European Journal of Immunology

Peer review correspondence

First Revision – authors' response – 16 January 2014

Reviewer: 1

Comments to the Author

This paper by Deaton et al. reported that IFNγ/IL-4 double-producing CD4 T cells are generated after S. mansoni infection, the DNA methylation signature at the ifng and il4 gene loci of the IFNγ+IL-4+ CD4 T cells is distinct from that of IFNγ or IL-4 single-producing cells, the level of Gata3 expression in IFNγ+IL-4+ cells is intermediate compared to that of Th2 and Th1 cells and that the level of Gata3 expression negatively correlates with the DNA methylation at the CpG island (CGI) on the gata3 gene locus in IFNγ+IL-4+ CD4 T cells. The authors suggest that the modification of intragenic CGI methylation is a new mechanism of cytokine gene regulation in effector CD4 T cells. Although their findings are interesting, several major points need to be addressed:

(1) In Figure 1, the authors should carefully analyze the population of IFNγ+IL-4+ CD4 T cells after S. mansoni infection. There is a possibility that the IFNγ+IL-4+ CD4 T cells might be doublet cells or dead cells, because these cells are on the diagonal line in Figure 1B. The authors should test the population of IFNγ+IL-4+ CD4 T cells after removing doublet cells and dead cells by using the combination of FSC-W and SSC-W, and dead cell staining reagents.

Response: The data shown in Figure 1 was gated on FSC-A/SSC-A to remove dead cells, after which doublets were removed using FSC-W and FSC-A prior to gating on CD4. This information has now been added to the legend for Figure 1 and included as Supplemental Figure S1. In addition, we have included a repeat experiment where a cell viability stain was used in Supplemental Figure S2. The IFNγ+IL-4+ population is still evident following exclusion of non-viable cells.

Note: To create Figure S1, we altered the gating on the CD4 population slightly to increase accuracy; this affected the IFNγ+IL-4+ populations, therefore Figure 1 has been updated accordingly.

(2) In Figures 2 and S1, the authors showed that the methylation of the il4, ifng and gata3 gene loci negatively correlated with the gene expression levels. Naïve CD4 T cells cannot produce more than the amount of IFNγ and IL-4 from Th1 and Th2 cells, respectively, so it is reasonable for naïve CD4 T cells to maintain the promoters of the il4 gene and CNS-6 of the ifng gene methylated. However, although naïve CD4 T cells are known to express Gata3, the gene body of Gata3 is methylated in naïve CD4 T cells, as well as in Th1 cells. This result does not fit their conclusions. The authors should discuss this discrepancy. It might be helpful to investigate the chromatin modifications, H3K4 methylation, H3K9 methylation and H3K14 acetylation at the target regions.

Response: We see that in cells isolated from S. mansoni infection DNA methylation of the Gata3 gene body CGI negatively correlates with gene expression levels. In naïve CD4+ T cells from uninfected

European Journal of Immunology

controls we find that this CGI is heavily methylated, even though there are reports in the literature that naïve cells express low levels of Gata3. We believe this does not invalidate the conclusion that, in an infection setting, there is a negative correlation between Gata3 expression and methylation. We have added a line to the text qualifying that we see the association observed between Gata3 gene body CGI methylation and transcriptional repression during infection (page 8, paragraph 2).
Interestingly, others have carried out analysis of chromatin modifications at Gata3 in naïve CD4 T cells and find that the chromatin environment is suggestive of gene repression (Wei et al., 2009). While we agree with the reviewer that further in depth analysis of the chromatin and methylation landscape of naïve CD4+ T cells would be of great interest, we think this beyond the scope and space constraints of our current study which focuses on IFNγ+IL-4+ T cells isolated from an infection setting.

(3) In Figure 3C, the authors separated IFNγ+IL-4+ CD4 T cells into two types of cells based on the Gata3 expression level; high and low. In Figure 3D, they showed that the methylation signature at the CGI of the gata3 gene locus in Gata3-high cells was lower than that in Gata3-low cells. The authors should reanalyze the Gata3 expression and IL-4/IFNγ production, and show whether the level of Gata3 expression correlates positively with IL-4 and negatively with IFNγ.

Response: As suggested, we have analysed IFNγ and IL-4 expression in the Gata3 high and Gata3 low populations and now include this data as Supplemental Figure S5. As expected, Gata3-high cells show a higher level of IL-4 expression and a lower level of IFNγ expression than Gata3-low cells. We have added a sentence describing these findings to the text (page 8, paragraph 2).

Reviewer: 2
Comments to the Author
Deaton et al. describe IFN-g/IL-4 co-producing CD4 T cells in spleens of S. mansoni-infected mice. These cells exhibit distinct DNA methylation levels at the Ifng, Il4 and Gata3 loci in between those of IFN-g+IL-4- and IFN-g-IL-4+ cells from the same mice. The IFN-g+IL-4+ population contains GATA-3 low and high cells. GATA-3 low cells display stronger DNA methylation than GATA-3 high cells at intragenic but not promoter sites of the Gata3 gene, suggesting that intragenic DNA methylation may be indicative of transcription levels.
The intermediate DNA methylation levels at key Th1 and Th2 gene loci in IFN-g+IL-4+ cells are an interesting correlative observation. It may reflect one mechanism that underlies the stable phenotype of this IFN-g+IL-4+ T cell population. However, the current manuscript has several aspects that require major revisions:

European Journal of Immunology

1.) Why do the authors claim that their finding of IFN-g+IL-4+ T cells upon infection with S. mansoni is "surprising" (p. 5, l. 15)? It should be mentioned that this phenotype has already been described [1-3], also in response to S. mansoni [4] and other helminth infections [5].

References (from reviewer)

1. Kelso A, Gough NM (1988) Coexpression of granulocyte-macrophage colony-stimulating factor, gamma interferon, and interleukins 3 and 4 is random in murine alloreactive T-lymphocyte clones. Proc Natl Acad Sci U S A 85: 9189-9193.

2. Paliard X, de Waal Malefijt R, Yssel H, Blanchard D, Chretien I, et al. (1988) Simultaneous production of IL-2, IL-4, and IFN-gamma by activated human CD4+ and CD8+ T cell clones. J Immunol 141: 849-855.

3. Firestein GS, Roeder WD, Laxer JA, Townsend KS, Weaver CT, et al. (1989) A new murine CD4+ T cell subset with an unrestricted cytokine profile. J Immunol 143: 518-525.

4. Lohning M, Grogan JL, Coyle AJ, Yazdanbakhsh M, Meisel C, et al. (1999) T1/ST2 expression is enhanced on CD4+ T cells from schistosome egg-induced granulomas: analysis of Th cell cytokine coexpression ex vivo. J Immunol 162: 3882-3889.

5. Peine M, Rausch S, Helmstetter C, Frohlich A, Hegazy AN, et al. (2013) Stable T-bet(+)GATA-3(+) Th1/Th2 Hybrid Cells Arise In Vivo, Can Develop Directly from Naive Precursors, and Limit Immunopathologic Inflammation. PLoS Biol 11: e1001633.

Response: In response to the reviewer's concerns, we have altered the text to better reflect previous work in this area. Relevant references have been added to the introduction (p3, paragraph 1), (Firestein et al., 1989; Kelso and Gough, 1988; Lohning et al., 1999; Paliard et al., 1988). In addition, we have referenced (Peine et al., 2013) again in the relevant part of the results section (p5, paragraph 1).

2.) In Fig. 1B, a control showing IFN-g vs. IL-4 production of PMA/ionomycin-restimulated CD4 cells from uninfected mice is required. These uninfected mice should be of a similar age as the infected animals to get an idea of the CD4 memory component independent of the infection with S. mansoni (cf. next point). The control without restimulation is not sufficient.

Response: We had omitted these data from the original submission to help reduce the size of the manuscript, in line with the E.J.I. short communication format. However, at the reviewer's suggestion, we now provide these data as Figure S2 which clearly shows that uninfected naïve controls display very few IFNγ+IL-4+ cells.

3.) The cells were neither restimulated in an antigen-specific manner nor taken from an effector organ such as the liver. Thus, the analyzed populations were rather undefined, most likely being a mixture of S. mansoni-specific effectors and other effector/memory cells with unknown differentiation history. This limitation should at least be critically discussed.

European Journal of Immunology

Response: Although the spleen is an accepted site for readout of the CD4+ T cell response during murine schistosome infection, we agree with the reviewer that it would be ideal to extend this work to include analysis of IFNγ+IL-4+ T cell populations in effector sites such as the liver or intestines. While we have preliminary data that hepatic IFNγ+IL-4+ CD4+ T cells can indeed be identified during murine schistosome infection, we have yet to begin characterisation of the methylation signature of these cells. Thus, timely generation of publishable data addressing this point is beyond the scope of the current resubmission.
As the reviewer has suggested, we have added the following text to the manuscript (page 6) to clarify this limitation of our study:
'During schistosome infection, the spleen is an important site for assessing responding lymphocytes, which include circulating effector and effector/memory CD4+ T cells (Taylor et al., 2009). An important next step in our studies will be to assess the methylation signature of IL-4+IFNg+ T cells isolated from effector sites such as the liver.'
Unfortunately, T cell restimulation with parasite antigens does not stimulate sufficient cytokine production to provide high enough resolution to clearly identify cytokine secreting CD4+ T cells by flow cytometry. For this technical reason, PMA/ionomycin is a standard and accepted technique for assessment of CD4+ T cell cytokine potential by flow cytometry during murine schistosome infection (Mentink-Kane et al., 2011; Pesce et al., 2009a; Pesce et al., 2009b). We eagerly await development of new reagents, such as functional MHC Class II tetramers, to enable more refined tracking of schistosome-specific CD4+ T cells. However, to our knowledge, such reagents are not yet available for study of schistosomes or any other helminth.

4.) A purity of 75%-96% is rather low for a FACS sort. May this explains why in Fig. 2A the Th1 cells showed strong demethylation at the Il4 gene compared with the naive control or may this result from their undefined differentiation history as discussed above? The authors should comment on this finding instead of just claim that “in conventional Th1 and Th2 cells only the locus for the signature cytokine lacked methylation” (p. 6, l. 23). The sort pictures should be included as a supplementary figure including the population that was only 75% pure to better define the contaminating cell fraction.

Response: To clarify this data, post-sort plots have been added as Figure S3. When calculating % purities in the original manuscript, we were quite conservative with our gating. For the revised submission, we have reanalysed the data with slightly less conservative gating, which has resulted in higher % purities. Irrespective, it is clear from the plots that the post-sort populations are quite distinct, and we would suggest that it is highly unlikely that the large differences in DNA methylation at the Il4 gene seen between Th1 and naïve cells (15% compared to 89% methylation) could be due to impure sorting.

5.) IFN-g+IL-4+ cells exhibit long-term co-expression of the Th1 and Th2 lineage-specifying transcription factors T-bet and GATA-3, respectively. Why do the authors only analyze DNA methylation of the Gata3 locus? Data on the Tbx21 locus should be included as well.

European Journal of Immunology

Response: The focus of the current manuscript is on CD4+ T cells isolated from a strongly Th2 polarised infection, and for this reason the emphasis of our investigation has been on Gata3. Tbx21 has a promoter CpG island but no gene body CpG island, in contrast to Gata3, and mining of existing CpG island DNA methylation data for naïve CD4 cells and in vitro differentiated Th1 and Th2 cells (Deaton et al., 2011) showed that the Tbx21 promoter CpG island is completely unmethylated in all of these cell types. This is consistent with the absence of methylation at promoter CpG islands more generally (Illingworth et al., 2010). Based on the absence of methylation observed for Tbx21 in naïve, Th1 and Th2 cells, we would suggest that it is highly unlikely that the DNA methylation status of Tbx21 changes in cells from S. mansoni infection. If the reviewer considers this a key discussion point, we would be happy to provide some text to clarify this in the manuscript, though space is somewhat limited.

6.) The selection of only one region at each gene for the DNA methylation analysis seems a bit arbitrary and requires explanation. The authors should at least show data for the promoter and one key regulatory element of each gene and maybe comment on further ones.

Response: As the amount of material recovered from sorts was limiting in these experiments, we chose to focus our efforts on a few key regions. For the Gata3 gene, assessment of methylation of the promoter CpG island and two regions of the gene body CpG island was carried out (3 regions total). For Ifng and Il4, the selection of regions for methylation analysis was based on published work on in vitro differentiated T cells (Schoenborn et al., 2007). For Ifng, we wanted to analyse a region that showed Th1-specific demethylation. CNS-6, which has been shown to be unmethylated in Th1 cells but heavily methylated in naïve and Th2 cells, fulfils such criteria. The Ifng promoter does not have these methylation characteristics and is unmethylated in both naïve and Th1 cells and only about 50% methylated in Th2 cells. Similarly, we wanted to analyse a region showing Th2-specific demethylation so therefore the Il4 promoter seemed like the most appropriate choice (Schoenborn et al., 2007).

7.) For Fig. 2 and Fig. 3, the results of the second experiment should be shown, perhaps as bar diagrams with error bars, normalized to the naive controls.

Response: At the reviewer's request, this information has been added to the revised manuscript as Figures 2D and 3E. In addition, Figure 3B has been edited to include data for repeats. Naïve controls are included on the bar graphs for Figure 2D, but infection samples have not been normalized to them, as we would suggest that in this case the raw figures allow more informative comparisons to be made.
Note: In addition, an error in the original submission was found in Figure 2B, where DNA methylation for Th2 cells was marked as 76%. This should have been 70%, which has been corrected in the revised manuscript.

European Journal of Immunology

8.) How do the authors interpret that the IFN-g+IL-4+ population contained a GATA-3 low compartment that shows GATA-3 expression levels and DNA methylation like Th1 cells? Does this contradict their claim of a distinct population?

Response: We would suggest that the IFNγ+IL-4+ cell population is distinct based on a number of features, not just Gata3 methylation. For example, unlike Th1 and Th2 cells, these cells co-express IFNγ and IL-4. In addition, they have a unique DNA methylation signature at the Ifng and Il4 genes.
It is interesting that the Gata3 low cells have Gata3 methylation levels similar to those of Th1 cells. However, the fact they express IL-4 makes them clearly different to Th1 cells. Perhaps variation in Gata3 methylation and expression levels within IFNγ+IL-4+ cell populations contributes to flexibility in the identity of these cells and allows them to possess both Th1 and Th2 characteristics, as we have suggested in the body of the manuscript (p8, paragraph 1).

9.) The site of the GATA-3 gene body CGI should be specified. Is it located in an exon?

Response: To clarify this point (shown in Figure 2C) we have added additional information to the text (p6, paragraph 2). The CGI overlaps the third exon of Gata3 but extends upstream of it.

10.) How is the differential DNA methylation at the GATA-3 gene body CGI related to the IFN-g/IL-4 co-expressing T cells? The authors should better connect these two parts.

Response: We would suggest that differential DNA methylation of the Gata3 gene body CGI is significant for two reasons.
Firstly, the differences we have identified in DNA methylation at Gata3 (as well as at Il4 and Ifng) between IFNγ+IL-4+ cells and conventional Th1 and Th2 cells shows that these cells are a distinct population, different to Th1 and Th2 cells on a molecular level.
Secondly, in IFNγ+IL-4+ cells the moderate levels of DNA methylation and Gata3 gene expression we have observed may represent an epigenetic state that allows for flexibility in the identity of these cells, allowing them to possess both Th1 and Th2 characteristics.
This is discussed on p7, paragraph 2 and additional discussion has been added to the text (p8, paragraph 1) to further emphasise this important issue.

References

Deaton, A.M., Webb, S., Kerr, A.R., Illingworth, R.S., Guy, J., Andrews, R., and Bird, A. (2011). Cell type-specific DNA methylation at intragenic CpG islands in the immune system. Genome research 21, 1074-1086.

European Journal of Immunology

Firestein, G.S., Roeder, W.D., Laxer, J.A., Townsend, K.S., Weaver, C.T., Hom, J.T., Linton, J., Torbett, B.E., and Glasebrook, A.L. (1989). A new murine CD4+ T cell subset with an unrestricted cytokine profile. J Immunol 143, 518-525.

Illingworth, R.S., Gruenewald-Schneider, U., Webb, S., Kerr, A.R.W., James, K.D., Turner, D.J., Smith, C., Harrison, D.J., Andrews, R., and Bird, A.P. (2010). Orphan CpG Islands Identify Numerous Conserved Promoters in the Mammalian Genome. PLoS genetics 6, e1001134.

Kelso, A., and Gough, N.M. (1988). Coexpression of granulocyte-macrophage colony-stimulating factor, gamma interferon, and interleukins 3 and 4 is random in murine alloreactive T-lymphocyte clones. Proceedings of the National Academy of Sciences of the United States of America 85, 9189-9193.

Lohning, M., Grogan, J.L., Coyle, A.J., Yazdanbakhsh, M., Meisel, C., Gutierrez-Ramos, J.C., Radbruch, A., and Kamradt, T. (1999). T1/ST2 expression is enhanced on CD4+ T cells from schistosome egg-induced granulomas: analysis of Th cell cytokine coexpression ex vivo. J Immunol 162, 3882-3889.
Mentink-Kane, M.M., Cheever, A.W., Wilson, M.S., Madala, S.K., Beers, L.M., Ramalingam, T.R., and Wynn, T.A. (2011). Accelerated and progressive and lethal liver fibrosis in mice that lack interleukin (IL)-10, IL-12p40, and IL-13Ralpha2. Gastroenterology 141, 2200-2209.

Paliard, X., de Waal Malefijt, R., Yssel, H., Blanchard, D., Chretien, I., Abrams, J., de Vries, J., and Spits, H. (1988). Simultaneous production of IL-2, IL-4, and IFN-gamma by activated human CD4+ and CD8+ T cell clones. J Immunol 141, 849-855.

Peine, M., Rausch, S., Helmstetter, C., Frohlich, A., Hegazy, A.N., Kuhl, A.A., Grevelding, C.G., Hofer, T., Hartmann, S., and Lohning, M. (2013). Stable T-bet(+)GATA-3(+) Th1/Th2 Hybrid Cells Arise In Vivo, Can Develop Directly from Naive Precursors, and Limit Immunopathologic Inflammation. PLoS biology 11, e1001633.

Pesce, J.T., Ramalingam, T.R., Mentink-Kane, M.M., Wilson, M.S., El Kasmi, K.C., Smith, A.M., Thompson, R.W., Cheever, A.W., Murray, P.J., and Wynn, T.A. (2009a). Arginase-1-expressing macrophages suppress Th2 cytokine-driven inflammation and fibrosis. PLoS pathogens 5, e1000371.

Pesce, J.T., Ramalingam, T.R., Wilson, M.S., Mentink-Kane, M.M., Thompson, R.W., Cheever, A.W., Urban, J.F., Jr., and Wynn, T.A. (2009b). Retnla (relmalpha/fizz1) suppresses helminth-induced Th2-type immunity. PLoS pathogens 5, e1000393.

Schoenborn, J.R., Dorschner, M.O., Sekimata, M., Santer, D.M., Shnyreva, M., Fitzpatrick, D.R., Stamatoyannopoulos, J.A., and Wilson, C.B. (2007). Comprehensive epigenetic profiling identifies multiple

distal regulatory elements directing transcription of the gene encoding interferon-gamma. Nature immunology 8, 732-742.

Taylor, J.J., Krawczyk, C.M., Mohrs, M., and Pearce, E.J. (2009). Th2 cell hyporesponsiveness during chronic murine schistosomiasis is cell intrinsic and linked to GRAIL expression. The Journal of clinical investigation 119, 1019-1028.

Wei, G., Wei, L., Zhu, J., Zang, C., Hu-Li, J., Yao, Z., Cui, K., Kanno, Y., Roh, T.Y., Watford, W.T., et al. (2009). Global mapping of H3K4me3 and H3K27me3 reveals specificity and plasticity in lineage fate determination of differentiating CD4+ T cells. Immunity 30, 155-167.

Second Editorial Decision – 10 February 2014

Dear Dr. MacDonald,

Thank you for submitting your revised manuscript ID eji.201344098.R1 entitled "A unique DNA methylation signature defines a population of IFNγ/IL-4 double positive T cells during helminth infection." to the European Journal of Immunology. Your manuscript has been re-reviewed and the comments of the referees are included at the bottom of this letter.

Although the referees have recommended publication, some revisions to your manuscript have been requested. Therefore, I invite you to respond to the comments of the referees and revise your manuscript accordingly.

If the revision of the paper is expected to take more than three months, please inform the editorial office. Revisions taking longer than six months may be assessed by new referees to ensure the relevance and timeliness of the data.

Once again, thank you for submitting your manuscript to European Journal of Immunology. We look forward to receiving your revision.

Yours sincerely,
Karen Chu

on behalf of Prof. Shimon Sakaguchi

Dr. Karen Chu

European Journal of Immunology

Peer review correspondence

Editorial Office
European Journal of Immunology
e-mail: ejied@wiley.com

www.eji-journal.eu

****

Reviewer: 1

Comments to the Author
The revision is satisfactory for this reviewer.

Reviewer: 2

Comments to the Author
In the revised version, the authors have addressed most of the raised concerns with sufficient modifications. However, in some minor points the manuscript still needs to be improved.

1. I still think that it is not correct to state that "in conventional Th1 and Th2 cells only the locus for the signature cytokine lacked methylation while the locus for the opposing cytokine was more extensively methylated" (p. 6, lines 9-11). The drastic demethylation of the IL-4 promoter in Th1 cells compared with the naive controls as shown in Fig. 2A is unexpected and also in contrast to the cited literature (Schoenborn et al., 2007). This should not be simply ignored but at least mentioned or better briefly discussed.

2. The authors do not provide data on the Tbx21 locus. However, as T-bet is the Th1 lineage-determining transcription factor, it should at least be part of the discussion with regard to the molecular mechanisms that may underlie the mixed Th1 and Th2 characteristics of the IFN-g+IL-4+ cells. If the authors do not expect differential demethylation of the Tbx21 locus between their cell populations, they should briefly mention why. But even if so, one would expect differential T-bet expression between these populations. T-bet expression by the IFN-g+IL-4+ cells but not by the IFN-g-IL-4+ cells may be an additional explanation why the former combine Th1 and Th2 properties.

3. In Fig. S2A the authors provide an IFN-g/IL-4 staining of CD4 T cells from the spleens of uninfected mice. This is an important control since the cells used in this study were not analyzed for their antigen

European Journal of Immunology

specificity. Therefore, the lower left dot plot in Fig. S2A should be included in the main Fig. 1B. It could replace the dot plot with the unstimulated cells which is not very informative.

Second Revision – authors' response – 14 February 2014

Reviewer 2 Comments to the Author:
In the revised version, the authors have addressed most of the raised concerns with sufficient modifications. However, in some minor points the manuscript still needs to be improved.
1. I still think that it is not correct to state that "in conventional Th1 and Th2 cells only the locus for the signature cytokine lacked methylation while the locus for the opposing cytokine was more extensively methylated" (p. 6, lines 9-11). The drastic demethylation of the IL-4 promoter in Th1 cells compared with the naive controls as shown in Fig. 2A is unexpected and also in contrast to the cited literature (Schoenborn et al., 2007). This should not be simply ignored but at least mentioned or better briefly discussed.

Response: The following discussion has now been added to the text (page 6).
"However, it is worth noting that in Th1 cells the Il4 promoter showed a dramatic decrease in DNA methylation compared to naïve controls (Figure 2A). This could suggest that demethylation of the Il4 locus is a general feature of CD4+ T cells in Th2 environments."

2. The authors do not provide data on the Tbx21 locus. However, as T-bet is the Th1 lineage-determining transcription factor, it should at least be part of the discussion with regard to the molecular mechanisms that may underlie the mixed Th1 and Th2 characteristics of the IFN-g+IL-4+ cells. If the authors do not expect differential demethylation of the Tbx21 locus between their cell populations, they should briefly mention why. But even if so, one would expect differential T-bet expression between these populations. T-bet expression by the IFN-g+IL-4+ cells but not by the IFN-g-IL-4+ cells may be an additional explanation why the former combine Th1 and Th2 properties.

Response: The following discussion has now been added to the text (page 9, paragraph 2).
"Tbx21, the locus from which T-bet is expressed, has a promoter CGI but no gene body CGI [15]. Analysis of existing DNA methylation data for naïve CD4+ cells and in vitro differentiated Th1 and Th2 cells showed that the Tbx21 promoter CGI is completely unmethylated in all of these cell types ([16], and data not shown). Based on this, as well as the lack of methylation at the Gata3 promoter (Supporting Information Figure 3C), we would suggest that it is unlikely that the DNA methylation status of Tbx21 changes in CD4+ T cells from S. mansoni infection. However, it is possible that expression of T-bet along with expression of Gata3 contributes to the dual identity of IFN-γ+IL-4+ cells, even if it is not associated with differences in DNA methylation."

3. In Fig. S2A the authors provide an IFN-g/IL-4 staining of CD4 T cells from the spleens of uninfected mice. This is an important control since the cells used in this study were not analyzed for their antigen specificity. Therefore, the lower left dot plot in Fig. S2A should be included in the main Fig. 1B. It could replace the dot plot with the unstimulated cells which is not very informative.

Response: Figure 1 has now been replaced with data from an experiment where CD4+ T cells enriched from both uninfected and infected animals were analysed. As a result Supporting Figure 2 been removed and Supporting Figure 1 now shows the gating for the experiment in Figure 1B which was done using live cell staining. For Figure 1B CD4 cells were selected using Dynal beads and this has been added to the Materials and Methods (p11).

Additionally, we have moved sort plots of Gata3 high and Gata3 low cells (Supporting Figure 3B in last draft) to Supporting Figure 4A so that the figure number reflects where the data is referred to in the text.

Third Editorial Decision – 19 February 2014

Dear Dr. MacDonald,

It is a pleasure to provisionally accept your manuscript entitled "A unique DNA methylation signature defines a population of IFN-γ/IL-4 double positive T cells during helminth infection." for publication in the European Journal of Immunology. For final acceptance, please follow the instructions below and return the requested items as soon as possible as we cannot process your manuscript further until all items listed below are dealt with.

Please note that EJI articles are now published online a few days after final acceptance (see Accepted Articles: http://onlinelibrary.wiley.com/journal/10.1002/(ISSN)1521-4141/accepted). The files used for the Accepted Articles are the final files and information supplied by you in Manuscript Central. You should therefore check that all the information (including author names) is correct as changes will NOT be permitted until the proofs stage.

We look forward to hearing from you and thank you for submitting your manuscript to the European Journal of Immunology.

Yours sincerely,
Karen Chu

on behalf of Prof. Shimon Sakaguchi

Peer review correspondence

European Journal of Immunology

Dr. Karen Chu
Editorial Office
European Journal of Immunology
e-mail: ejied@wiley.com
www.eji-journal.eu
